# Confined microemulsions: pore diameter induced change of the phase behavior†

**DOI:** 10.1039/d4ra01283f

**Published:** 2024-04-19

**Authors:** René Haverkamp, Margarethe Dahl, Tim Julian Stank, Jessica Hübner, Peter Strasser, Stefan Wellert, Thomas Hellweg

**Affiliations:** a Department of Physical and Biophysical Chemistry, University of Bielefeld Universitätsstraße 25 Bielefeld 33615 Germany thomas.hellweg@uni-bielefeld.de; b Stranski-Laboratorium für Physikalische und Theoretische Chemie, Technical University of Berlin Straße des 17. Juni 124 Berlin 10623 Germany s.wellert@tu-berlin.de; c Department of Chemistry, Chemical Engineering Division, Technical University of Berlin Straße des 17. Juni 124 Berlin 10623 Germany

## Abstract

In the present work, the temperature-dependent phase behavior of a C_10_E_4_ based microemulsion is studied in different meso–macroporous glasses, as a function of their pore diameter. The phase behavior in these pores is investigated by small-angle X-ray scattering (SAXS). The crucial parameter we discuss based on the SAXS results is the domain size of the bicontinuous phase. Using a simplified model to fit the scattering data, we can observe the microemulsion inside the pores. These experiments reveal a temperature-dependent change in domain sizes of the bicontinuous microemulsion only for large pores.

## Abstract 2

Possible temperature-dependent structure of the microemulsion within the pores of controlled pore glasses. Small pore sizes seem to affect the temperature-dependent phase behavior of the microemulsion.

## Graphical abstract

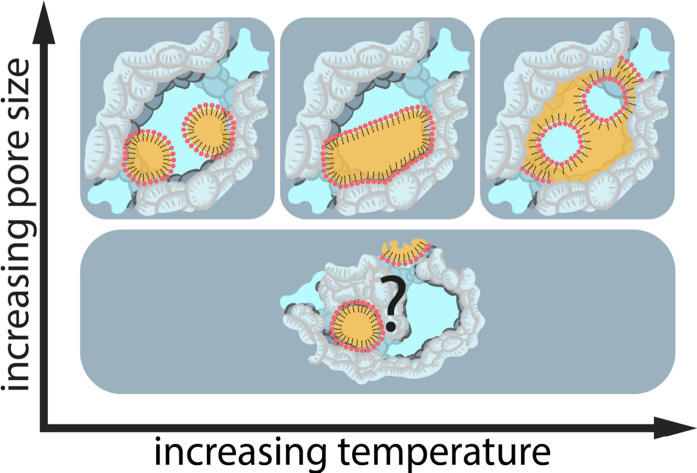

## 1 Introduction

Microemulsions are thermodynamically stable mixtures of oil and water that exhibit a complex phase behavior.^1–8^ Their unique properties have led to applications in enhanced oil recovery (EOR),^9–11^ decontamination,^12–14^ cosmetics,^15^ drug delivery,^16–18^ nanoparticle fabrication,^19^ and catalysis.^20^ Due to the growing demand for EOR applications worldwide, there are many studies aiming at enhancing efficiency and reduce the environmental impact of microemulsion flooding.^21–23^ In our study, we use microemulsions based on alkyloligoethyleneglycol surfactants, because of their relative nontoxicity and low impact of the salt concentration on the phase behavior.^24^ These surfactants show a pronounced temperature-dependent phase behavior.^25^ This is why the phase behavior of these microemulsions can be studied regarding their composition and temperature.^26^ To reduce complexity, many studies focus on equal volume fractions of oil and water and then investigate the phase behavior as a function of temperature and surfactant concentration. This approach leads to the Kahlweit-fish cut of the phase prism. Under certain conditions, an isotropic, single-phase region, the bicontinuous phase, is obtained.^26^ The geometry of oil and water within this structure is bicontinuous, and the interfacial tension between the oil and water phases reaches its minimum in this temperature range.^27–29^ This is also why this phase is promising for cleaning, decontamination, or EOR applications.^30–32^ However, in these applications, understanding the bulk phase behavior can only serve as a base for understanding the actual processes in the system, because the confinement of the microemulsion by an interface must be considered. The interaction of a nonionic surfactant microemulsion with a flat surface has been studied in many works,^33–36^ including simulations,^37^ investigations under shear stress,^38^ and at high-pressure conditions.^39^ In the case of bicontinuous microemulsions, the main difference between the interface and the bulk structure is the planar geometry near an interface, which becomes sponge-like when the distance to the interface increases. Since, in many relevant applications, the surface is rough or even porous, we must also consider the influence of curved interfaces. For example, water confined in a porous solid smaller than 2 nm will not form ice.^40^ The binary isobutyric acid–water solution shows an extended temperature-dependent phase transition, exhibiting a lowered upper critical solution temperature when confined in a controlled pore glass.^41,42^ Concerning ternary microemulsions, a paper by Prause *et al.* shows the arrangement of microemulsion droplets in pores^43^ and concludes that the microemulsion droplets have a bulk-like size when the diameter of the droplets is smaller than the pore diameter and that they are elongated cylinders when the size of the microemulsion droplets is larger than the pore diameter. However, at least to our knowledge, the temperature-dependent phase behavior of a bicontinuous tetraethyleneglycol–monodecylether (C_10_E_4_) microemulsion in a hydrophilic pore system has not yet been studied. This study aims at investigating the general phase behavior of microemulsions confined in porous glasses by investigating the domain sizes over a large temperature and pore size range.

## 2 Experimental

### 2.1 Materials

Water was taken from a Sartorius Arium VF pro ultrapure water system (Göttingen, Germany). Tetraethyleneglycol–monodecylether C_10_E_4_ (>95.0%) was obtained from Bachem, and *n*-octane (>99.0%) was obtained from Honeywell. The controlled pore glasses (CPG) used in this work were obtained from PolyOrg, Inc. The nominal pore diameters of the CPG studied were 1000 Å, 500 Å, 184 Å. To purify the CPG, they were stored in concentrated H_2_SO_4_ solution for two days and then rinsed with ultrapure water using a Soxhlet extractor. To test for remaining SO_4_^2−^, the samples were washed with water, then tested for remaining SO_4_^2−^ anions using a BaCl_2_ solution. After no SO_4_^2−^ could be detected, the CPG was dried overnight at 80 °C.

### 2.2 Sample preperation

To characterize ternary microemulsions, the weight fraction of the surfactant *γ* and the relative volume ratio of oil and water *α* are useful comparators, regardless of the total volume of the batch.1
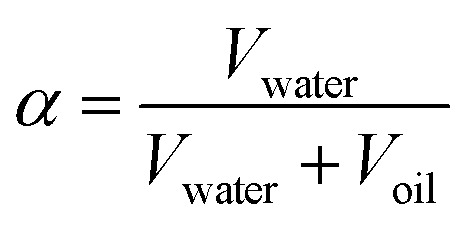
2
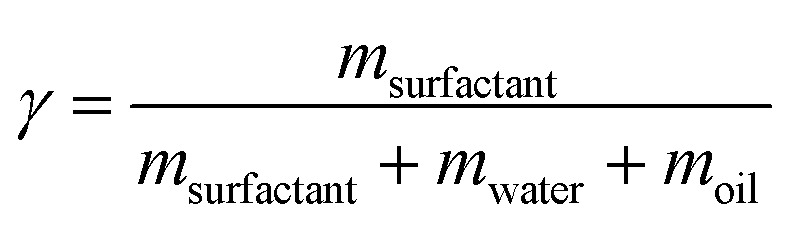


The microemulsion (ME) used was prepared and tempered at 22 °C and consisted of C_10_E_4_/water/*n*-octane with *α* = 0.5 and *γ* = 0.14. The complete formation of a bicontinuous phase at 20 °C was confirmed by a faint blue translucent sample that showed birefringence only under shear when observed under a microscope with crossed polarizers. For the subsequent SAXS experiments, 1 mm quartz capillaries from WJM-Glas Müller GmbH are used as sample cells. They were filled with the microemulsion, and the CPG was slowly added until a 5 mm supernatant of the microemulsion was left. Throughout the process, the turbidity of the microemulsion did not increase, indicating a stable microemulsion after adding the porous solid. The prepared quartz capillary was sealed and transferred to a temperature-controlled SAXS sample holder to perform SAXS measurements.

## 3 Methods

### 3.1 Porosimetry

To obtain the pore diameter *Ø*_Hg,BET_ of the CPGs (Table 1), Hg- and N_2_-porosimetry were performed. The Hg-intrusion measurements were performed for CPG1000 and CPG500 by the Bundesanstalt für Materialforschung und -prüfung. For CPG184, N_2_-physisorption isotherms were obtained at 77 K using an Autosorb-1 (QUANTACHROME). To minimize the dead volume, the used glass tubes were also filled with a glass rod and glass wool. The samples were degassed under vacuum at 90 °C for at least 24 h to remove any water and gas adsorbates. Brunauer–Emmett–Teller (BET) method in a pressure range of *p*/*p*_0_ = 0.04 to 0.2 was applied to calculate the overall surface area using a multipoint fit. The Barrett–Joyner–Halenda (BJH) method was applied to calculate the pore size distribution. A detailed description of these methods can be found in the literature.^44^

**Table 1:** Fit results and porosity data of CPG-1000, -500, -184. The parameters of the Teubner–Strey model are *d*, the domain size, *ξ*, the correlation length, and *B*, a scaling factor. The pore diameters resulting from SAXS *Ø*_SAXS_ are given by a multiplication of the porosity with the domain size *d*

CPG/Å	*d*/Å	*ξ*/Å	*B*	Porosity/%	*Ø*_SAXS_/Å	*Ø*_Hg,BET_/Å
1000	1353	507	2.325 × 10^6^	77	1038	1116
500	625	313	5.550 × 10^5^	72	445	491
184	381	79	8.581 × 10^3^	66	251	157

### 3.2 Small-angle X-ray scattering (SAXS)

Small-angle X-ray scattering is a well-established method to obtain information about the structure of microemulsions. Hence, using SAXS to obtain information about the microemulsion inside the pores seems straightforward. SAXS measurements were performed using an in-house SAXS/WAXS system (XEUSS, Xenocs, Sassenage, France). Radial integration of the 2D detector image recorded by a Pilatus 300K hybrid pixel detector (Dectris, Baden Deattwil, Switzerland) was performed using the Foxtrot software.^45^ To obtain the intensity of the scattered beam on an absolute scale, background correction and normalization with glassy carbon type 2 (ref. 46) were performed. In small-angle X-ray scattering, the intensity of a diffracted X-ray beam is determined as a function of the scattering vector *q*. The scattering vector, which depends on the scattering angle θ and the wavelength of light *λ*, is given as follows:3
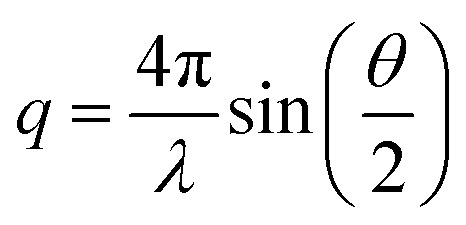


The scattering signal from controlled porous glasses can be modeled in several ways.^47,48^ In general, CPGs also have a bicontinuous, sponge-like structure.^49^

Despite the scattering of the porous matrix, two distinct scattering contributions can be observed when the microemulsion is inside the pores. One is at *q* values smaller than the signal of the CPG, and one is at higher *q* values than the signal of the CPG. It has been shown that the scattering contribution from randomly distributed binary fluid domains leads to a quadratic Lorentzian scattering behavior.^50,51^ Hence, to model the scattering contribution of these regions at small *q* values, the quadratic Lorentzian term in eqn (4) is used. The Lorentzian we use has *A* as the scaling factor, *ξ*_L_ as the Lorentzian correlation length, and *n* as the Lorentzian exponent. The parameter *q*_0_ is the peak position of the Lorentzian.4
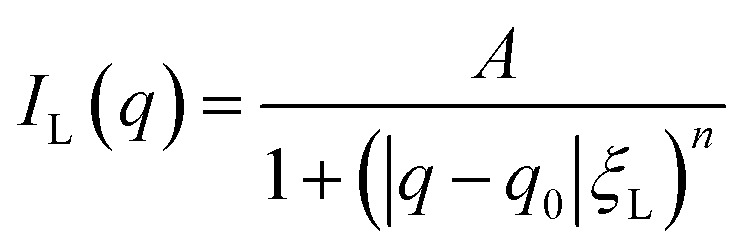


The relation *d*_Lz_ = 2π/*q*_0_ can be used to find the average center-to-center distance of microphase-separated structures, while the Lorentzian screening length is obtained from the size of the structures. The Lorentzian exponent is set to *n* = 4.

The Teubner–Strey model provides a simple model that describes the scattering of the bicontinuous phase around the maximum *q*_max_ of the correlation peak.^52^ High *q* values can be fitted if the diffuse surfactant layer is considered.^53^ The scattering intensity of the Teubner–Strey model *I*_TS_(*q*) scales with the factor *B* in which the volume fraction of a phase, the scattering contrast, and the correlation length *ξ* are considered. However, because these parameters cannot be accurately determined inside the porous solid, *B* is used only as a scaling factor.5
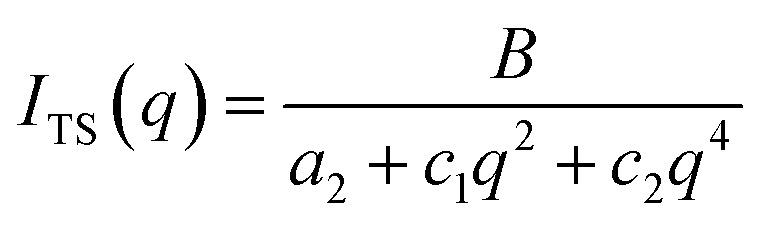


The parameters *a*_2_, *c*_1_, and *c*_2_ yield the periodicity of the oil and water domains, *i.e.* the domain size *d*,6
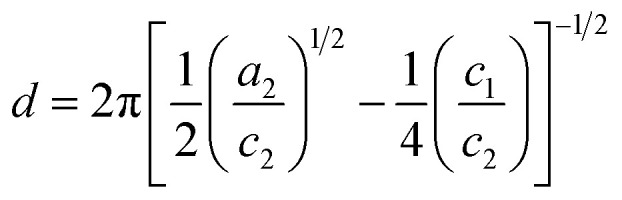
and the correlation length *ξ* measures the distance between a domain and a random environment without a long-range structure. *ξ* can be interpreted as the decay length of the quasi-periodic structure.7
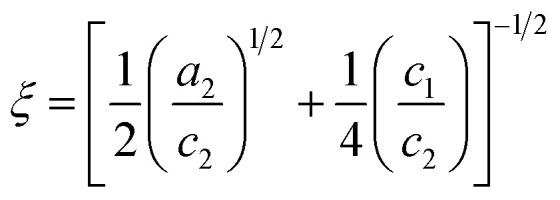


The parameters *a*_2_, *c*_1_, and *c*_2_ are defined by *d* and *ξ* using the following relations.8
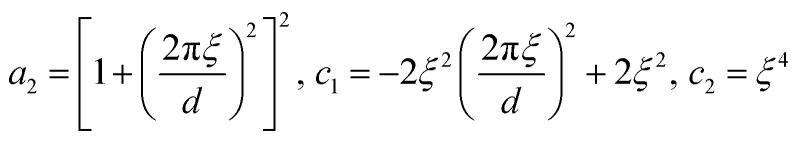


To model the complete signal of the microemulsion inside the CPG matrix, we use a linear combination of the signal from the CPG matrix, the Teubner–Strey model to account for the microemulsion signal, and a Lorentzian for possible phase-separated regions for data analysis. Note that the scattering contribution of the matrix is scaled by a factor *C* to account for different contrasts of the CPG matrix in the presence of the microemulsion. In total, this approach leads us to seven variable parameters (*A*, *B*, *C*, *d*, *ξ*, *q*_0_, *ξ*_L_), three of which are amplitude parameters (*A*, *B*, *C*).

9 *I*(*q*) = *C* × *I*(*q*)_CPG_ + *I*(*q*)_L_ + *I*(*q*)_TS_

## 4 Results and discussion

### 4.1 Bulk microemulsion

**Fig. 1:**
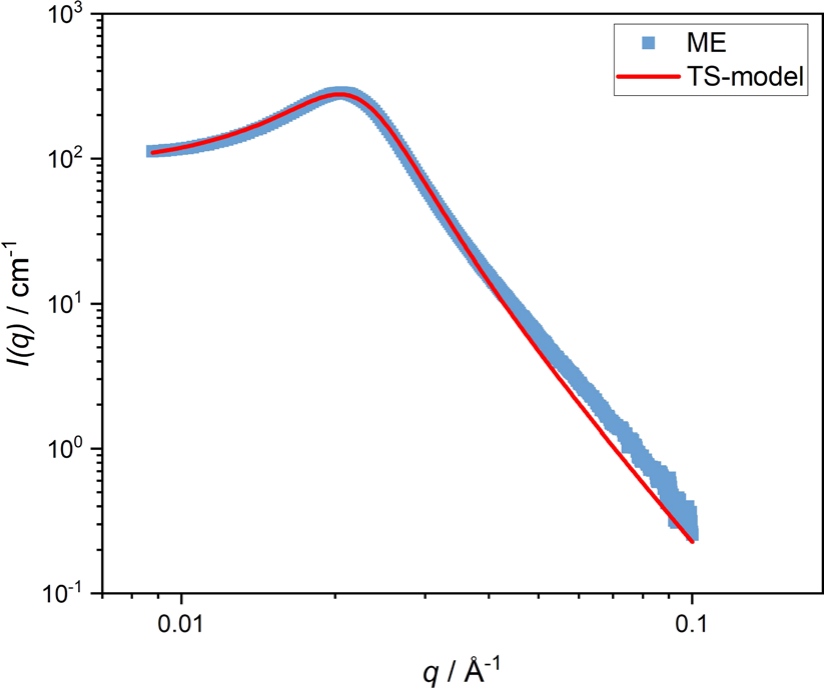
SAXS curves of a ternary C_10_E_4_/water/*n*-octane microemulsion at 20 °C. The fit to the Teubner–Strey model (eqn (5)) is shown as a red line.

Firstly, the bulk behavior of the microemulsion is examined as a reference. A temperature-dependent measurement of the bulk microemulsion at 20 °C with SAXS revealed a broad correlation peak at *q*_max_ = 0.031 Å^−1^ typical of a bicontinuous microemulsion, which can be described by the Teubner–Strey model (eqn (5), Fig. 1). The determined mean domain size *d* = 191 Å and a correlation length of *ξ* = 97 Å. Under the assumption that the oil and the water phases are isometric, we get a mean domain size of 95.5 Å for the oil and water domains.

### 4.2 Controlled pore glasses

**Fig. 2:**
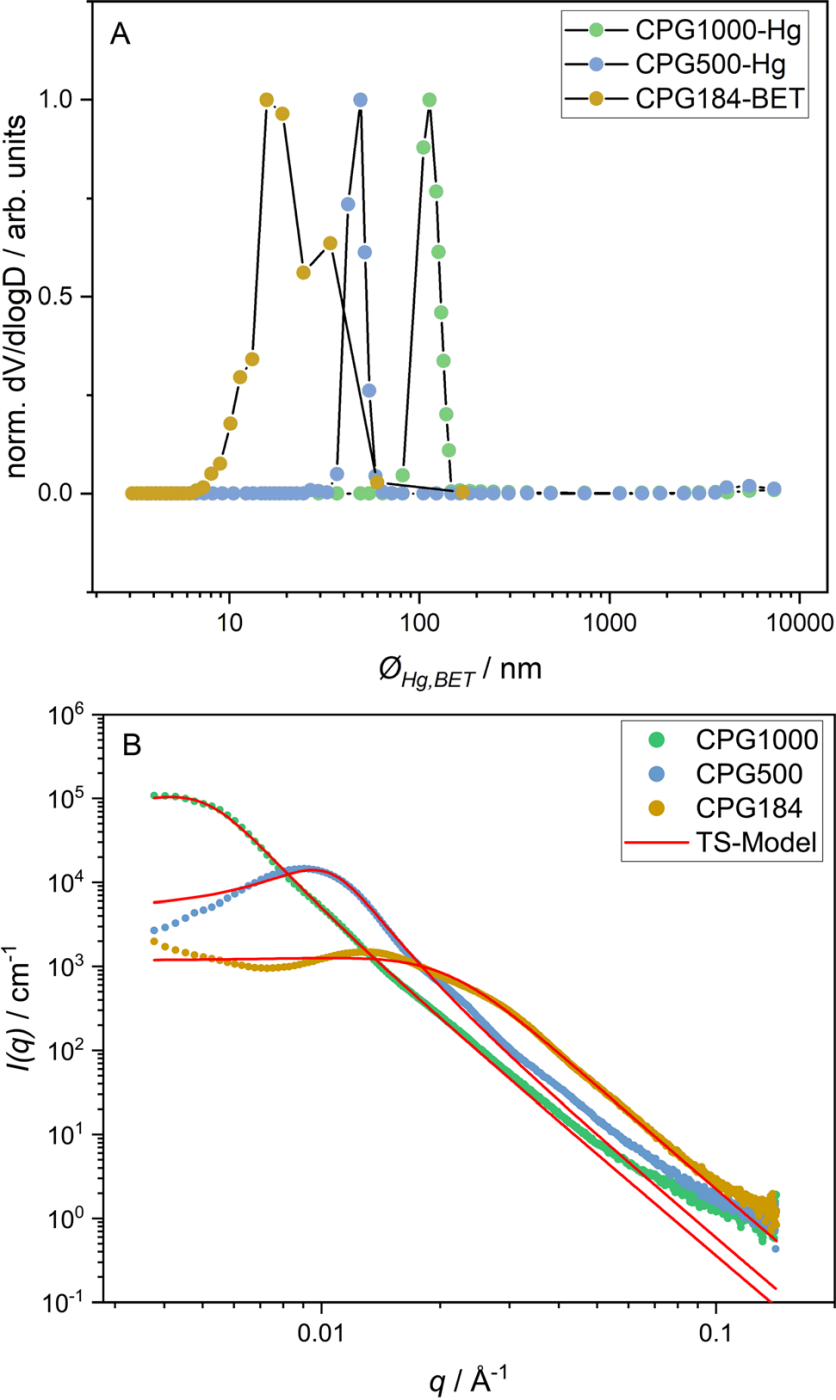
Porosimetry data in (A) and SAXS curves in (B) of CPG-1000, -500, and -184. The fit according to the Teubner–Strey model (eqn (5)) is shown as a red line (B).

We performed SEM measurements on all investigated CPGs to get a general impression of the real space structure. For the large structures of CPG1000, the bicontinuous character can be seen and is displayed in the ESI.† The Hg- and N_2_-porosimetry normalized pore size distribution of the CPGs is shown in Fig. 2A. We observed a broad pore size distribution for the *Ø*_BET_(CPG184) = 8 nm to 60 nm and a narrow distribution for *Ø*_Hg_(CPG500) = 37 nm to 59 nm and *Ø*_Hg_(CPG1000) = 82 nm to 143 nm. The measured mean pore diameters *Ø*_Hg,BET_ are close to the nominal pore diameters given by the supplier (Table 1).

The SAXS curves of the CPGs used in this work are shown in Fig. 2B. For the CPG500 and the CPG184, a broad scattering peak at *q*_max_ = 0.009 Å^−1^ and *q*_max_ = 0.013 Å^−1^ is observable. For the CPG1000, only about half of the main correlation peak is detected at *q*_max_ = 0.0045 Å^−1^. Additionally, a shoulder is present at *q* > *q*_max_. In this *q* range, the decay of the scattering signals follows a *q*^−3.8^ power law typical of a fractally rough pore surface, attributed in the literature to chemisorbed water clusters.^54^ At *q* < *q*_max_, an increased scattering intensity is observed for CPG184. Using USANS, Kim *et al.* demonstrated that mesopores could not be the reason for this increase, as the signal persists when the signal from the main correlation peak is matched out.^55^ A possible explanation for this signal is an incomplete leaching process during the CPG production, resulting in an inhomogeneous scattering length density of the solid phase.

A bimodal pore size distribution could be responsible for the shoulder at higher *q* values. Also, a fractal structure scattering^56^ and a long-range structure correlation leading to a second structure factor peak^55^ have been discussed earlier. For bicontinuous structures, multiple scattering effects can also lead to a shoulder in this region.^57^

The analysis of the scattering signal of CPG is not trivial, and there is not yet a complete model describing all its properties. Several ways of modeling the scattering curves of CPGs are presented in the literature.^47,48^ Since the structure of CPG is known to be bicontinuous, the Teubner–Strey model (eqn (5)) is chosen to fit the data to obtain information about the mean domain sizes of the CPG. However, there are deviations from the Teubner–Strey-like scattering, such as the pronounced shoulder at *q* > *q*_max_.

The results of the Teubner–Strey fit are shown as a red line in Fig. 2B. Table 1 lists the resulting fitting parameters. For the CPG1000 and CPG500, we observe a good fit to the scattering data in the peak region. However, the model fails partly to describe the scattering curve of CPG184. Indicating a bimodal pore size distribution, as seen in the porosimetry results in Fig. 2A.

### 4.3 Imbibed microemulsion

**Fig. 3:**
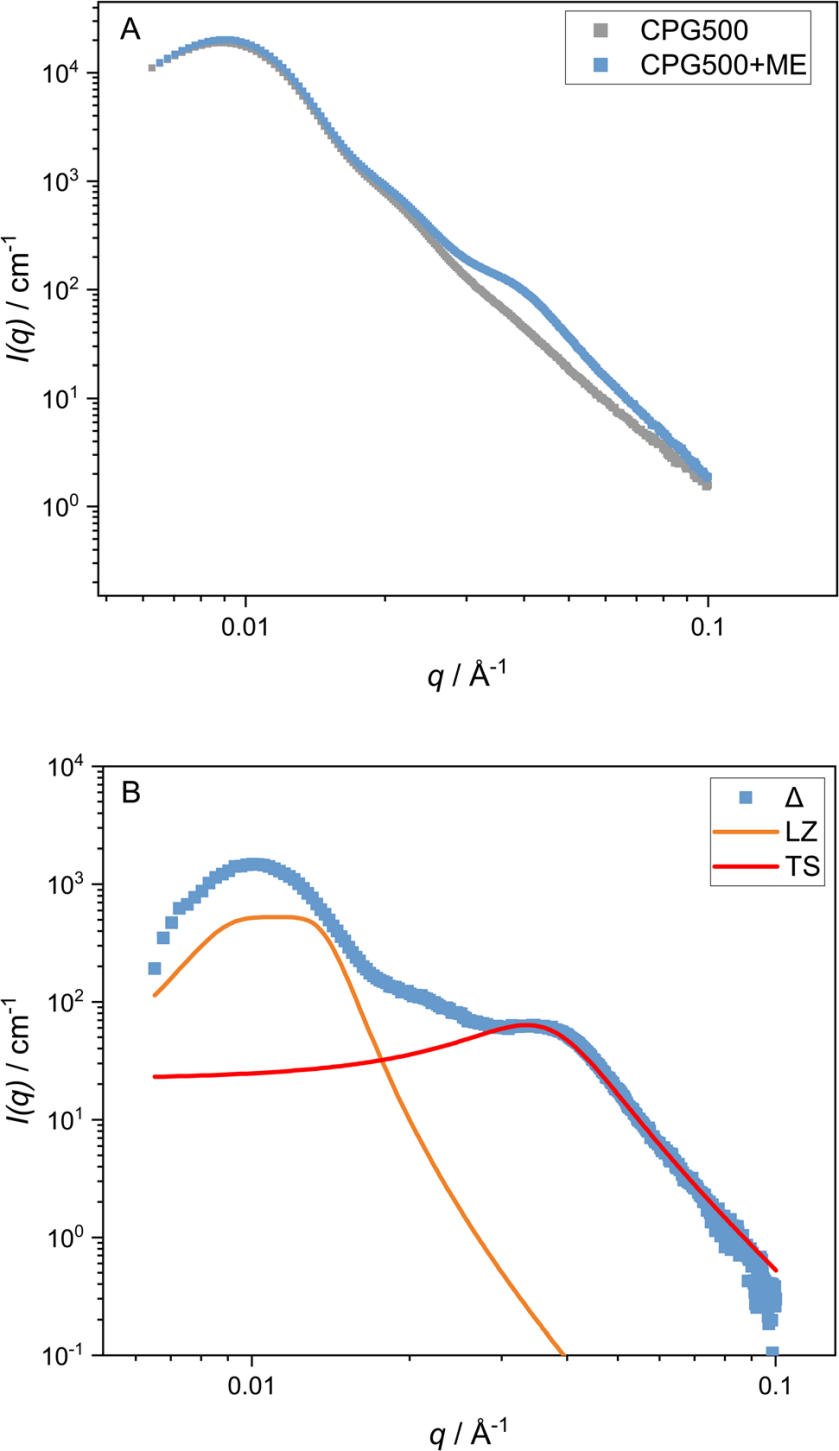
SAXS curves for CPG500 with and without the microemulsion (A), and the SAXS curve after subtracting the matrix signal from the signal of the soaked CPG (B). In addition, the respective contributions of the Lorentzian and the Teubner–Strey Model to the fit are displayed (B).

By comparison of the scattering signal of the matrix (Fig. 2) and the signal of the microemulsion imbibed in CPG (Fig. 3), a pronounced shoulder at *q* > *q*_max_ can be observed. After subtracting the matrix signal scaled by a factor, which is necessary because the contrast of the matrix changes after the pores are filled with the microemulsion, we can also observe another contribution at low *q* < *q*_max_. To fit both features, a combination of the matrix scattering signal, the Teubner–Strey model, and a Lorentzian peak for the contribution at *q* < *q*_max_ are employed as described in eqn (9). This simplification of the scattering signal can describe the signal of the microemulsion inside the CPG well enough to model the shoulder contribution of the microemulsion at high *q*-values. However, the model does not describe the contribution at lower *q* values, discussed in Section 3.2.

### 4.4 Temperature dependent behavior

#### 4.4.1 Microemulsion

**Fig. 4:**
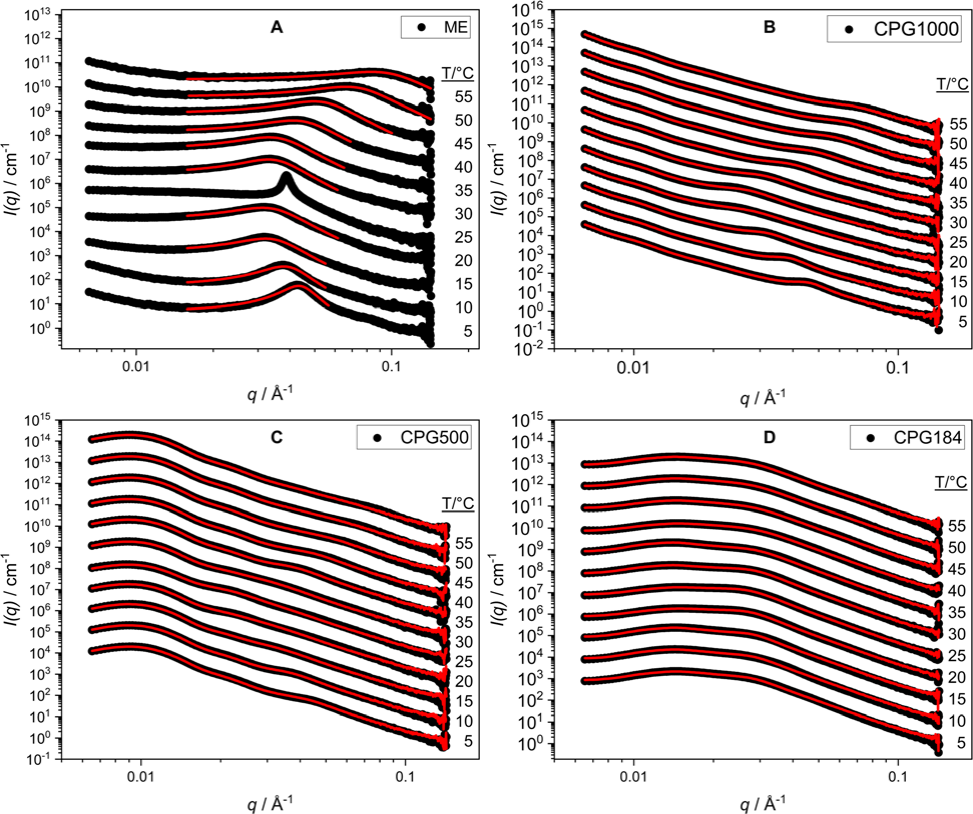
SAXS curves for the ME as bulk (A) and in CPG-1000 (B), -500 (C), and -184 (D) at temperatures from 5 °C to 55 °C. The fits according to eqn (9) are shown in red.

The temperature-dependent phase behavior was measured using the same protocol as in Section 4.3, measuring in 5 °C steps in the temperature range of 5 °C to 55 °C. The results show a temperature-dependent shift of the correlation peak position (Fig. 4A). At low temperatures, the microemulsion consists of two phases, with a water continuous oil-in-water emulsion in the lower phase.^58^ The SAXS data of the water phase show a strong correlation peak. With increasing temperature, the peak shifts to smaller *q* values, and the shape of the peak broadens. At temperatures between 19 °C and 31 °C, the single-phase region is observed in the bulk sample. Here, the typical scattering pattern of a bicontinuous microemulsion is present. At 25 °C, we measure a sharp correlation peak showing the existence of the lamellar phase at this temperature (Fig. 4A). Upon heating above temperatures where the single-phase region is stable, we obtain a two-phase region with an oil continuous water-in-oil emulsion in the upper phase.^58^ Heating to 55 °C shows the opposite trend observed for the water phase at low temperatures. With increasing temperature, the correlation peaks shift to higher *q* values. The shift in the correlation peak position as a function of temperature agrees well with the expectation of a temperature-dependent curvature of the microemulsion based on alkyloligoethylene surfactants. At low temperatures, there is a strong curvature of the surfactant interface towards the oil phase. With increasing temperature, the curvature decreases until a bicontinuous structure is formed, exhibiting a locally flat interface with zero mean curvature. A further increase in temperature leads to a negative curvature of the surfactant interface, and inverse water-in-oil droplets are formed.

#### 4.4.2 Controlled pore glass imbibed with microemulsion

**Fig. 5:**
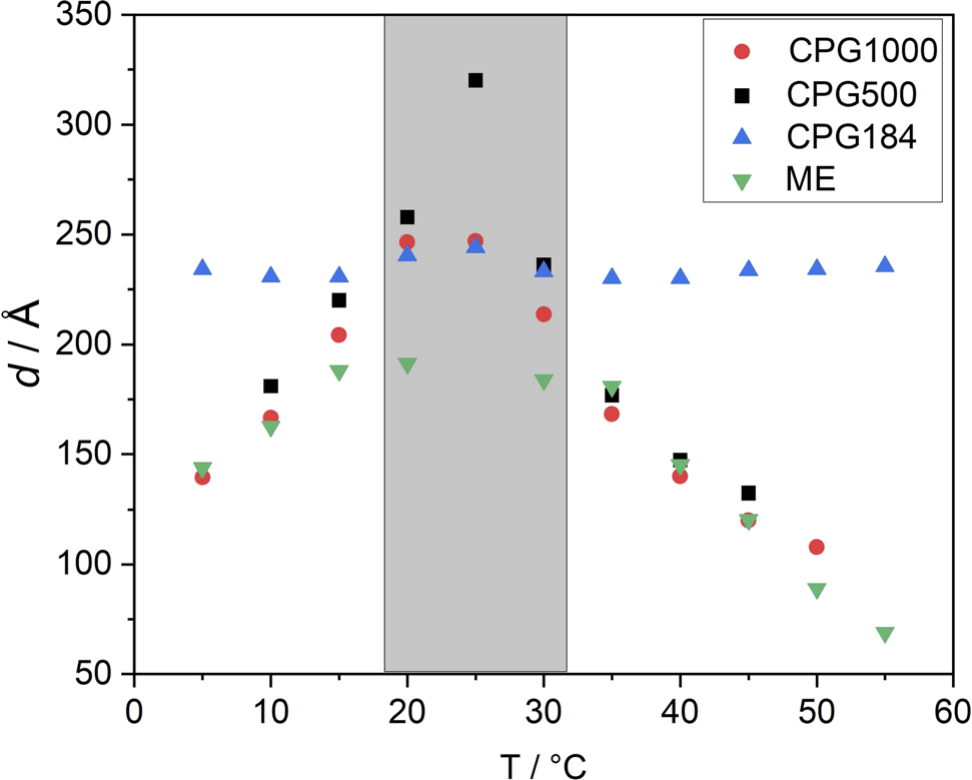
Domain size for ME as bulk and soaked in the CPG-1000, -500, -184 according to eqn (6). The parameters shown here result from a fit to the corresponding SAXS data displayed in Fig. 4 based on eqn (9). The temperature range with a stable one-phase region of the bulk ME is indicated in grey.

We observed no macroscopic phase separation at all measured temperatures for the imbibed microemulsion. All parameters of the fitted model (eqn (9)) are displayed in the ESI.† The main parameter of interest is the domain size *d* of the microemulsion shown in Fig. 5. We can observe the temperature-dependent variation of the domain sizes of the bulk phase microemulsion and the embedded microemulsion in CPG1000 and CPG500, shown as green, red, and black data points in Fig. 5. However, this behavior no longer appears favorable for the small pore diameters of CPG184, as blue data points in Fig. 5. Here, we observe an approximately constant domain size around *d* = 230 Å as a function of temperature. In our approach, bulk-like structures can be formed and are measured in SAXS for large pore diameters, leading to the observed change in domain size. The domain size of the microemulsion in CPG184 remains constant over the entire temperature range. As the main correlation peak of the CPG184 and the microemulsion are very close, we are probably unable to resolve the microemulsion's scattering contribution.

## 5 Conclusion

No macroscopic phase separation was observable in sample cells at any measured temperature in the range of 5 °C to 55 °C. This indicates that the two-phase region of the microemulsion, if present, is formed at the scale of the pore network. In regards to the domain size of the microemulsion, we have shown that the temperature-dependent phase behavior of the C_10_E_4_/water/*n*-octane microemulsion in a CPG resembles the bulk phase of the microemulsion for samples with large pore diameters. This hints at a low interaction of the CPG surface with the microemulsion. These results are desirable for applications such as decontamination and enhanced oil recovery. Low surfactant interaction with the pore surface is necessary to remove substances from the pores without losing the surfactant. These findings can potentially help address one of the main challenges of using microemulsions in EOR. Up-to-date approaches lead to high surfactant losses or include further manufacturing steps, like adding alcohols or inducing a salinity gradient, increasing the environmental impact.^59–62^

The water/oil interface does seem to alter the microemulsion domain size in small pores. This would be remarkable because it happens even if the domain sizes in the larger CPG pores and the bulk microemulsion would be much smaller than the pore diameter of the CPG184 with *d*_ME_ = 120 Å and pore diameter *Ø*_BET_ = 251 Å (Fig. 5) respectively. However, as the SAXS signal of the microemulsion and the SAXS signal of CPG184 overlap, this can also be an artifact. Further insights into the actual geometry inside the CPG remain unclear and require other methods to be used in future research on this system. Self-diffusion NMR, small-angle neutron scattering contrast variation,^43^ and neutron spin-echo experiments are our following research goals, as they have led to insights into binary fluids inside porous CPG.

## Conflicts of interest

The authors declare no conflict of interest.

## Supplementary Material

RA-014-D4RA01283F-s001

## References

[cit1] Kahlweit M., Strey R., Haase D., Kunieda H., Schmeling T., Faulhaber B., Borkovec M., Eicke H.-F., Busse G., Eggers F., Funck T., Richmann H., Magid L., Söderman O., Stilbs P., Winkler J., Dittrich A., Jahn W. (1987). J. Colloid Interface Sci..

[cit2] Strey R. (1994). Colloid Polym. Sci..

[cit3] Microemulsions: Background, New Concepts, Applications, Perspectives, Wiley, Chichester, West Sussex, 1st edn, 2009

[cit4] Fanun M. , Microemulsions, CRC Press, 2008

[cit5] Hellweg T. (2002). Curr. Opin. Colloid Interface Sci..

[cit6] Jolivalt C., Minier N., Renon N. (1989). Fluid Phase Equilib..

[cit7] Langevin D. (1992). Annu. Rev. Phys. Chem..

[cit8] Lu M., Lindman B., Holmberg K. (2024). Phys. Chem. Chem. Phys..

[cit9] Handbook of Microemulsion Science and Technology, ed. P. Kumar and K. L. Mittal, Taylor and Francis, London, 1st edn, 2017

[cit10] Mandal A. and Ojha K., Enhanced Oil Recovery, CRC Press, Boca Raton, 2023

[cit11] Santanna V. C., Curbelo F., Castro Dantas T. N., Dantas Neto A. A., Albuquerque H. S., Garnica A. (2009). J. Pet. Sci. Eng..

[cit12] Wellert S., Karg M., Imhof H., Steppin A., Altmann H.-J., Dolle M., Richardt A., Tiersch B., Koetz J., Lapp A., Hellweg T. (2008). J. Colloid Interface Sci..

[cit13] Wellert S., Imhof H., Dolle M., Altmann H.-J., Richardt A., Hellweg T. (2008). Colloid Polym. Sci..

[cit14] Decontamination of Warfare Agents: Enzymatic Methods for the Removal of B/C Weapons, ed. A. Richardt, Wiley-VCH, Weinheim, 1st edn, 2008

[cit15] Boonme P. (2007). J. Cosmet. Dermatol..

[cit16] Luo F., Zhong T., Chen Y., Guo Q., Tao L., Shen X., Fan Y., Wu X. (2023). Pharmaceutics.

[cit17] Shukla T., Upmanyu N., Agrawal M., Saraf S., Saraf S., Alexander A. (2018). Biomed. Pharmacother..

[cit18] Sharma A. K., Garg T., Goyal A. K., Rath G. (2016). Artif. Cells, Nanomed., Biotechnol..

[cit19] Ström L., Ström H., Carlsson P.-A., Skoglundh M., Härelind H. (2018). Langmuir.

[cit20] Schwarze M., Pogrzeba T., Volovych I., Schomäcker R. (2015). Catal. Sci. Technol..

[cit21] Kumar A., Saw R. K., Mandal A. (2019). Chem. Eng. Res. Des..

[cit22] Kaushik A., Joshi D., Kumar Saw R., Bala Rathi K., Mitra S., Mandal A. (2024). Fuel.

[cit23] Saw R. K., Rane P. M., Joshi D., Prakash S., Jangid L., Mandal A. (2023). J. Mol. Liq..

[cit24] Saw R. K., Sinojiya D., Pillai P., Prakash S., Mandal A. (2023). ACS Omega.

[cit25] Ontiveros J. F., Pierlot C., Catté M., Molinier V., Salager J.-L., Aubry J.-M. (2014). Colloids Surf., A.

[cit26] Stubenrauch C. , Microemulsions, John Wiley & Sons, Ltd, Chichester, UK, 2009

[cit27] Langevin D. (1992). Annu. Rev. Phys. Chem..

[cit28] Strey R. (1996). Curr. Opin. Colloid Interface Sci..

[cit29] Sottmann T., Strey R. (1997). J. Chem. Phys..

[cit30] Zhu T., Kang W., Yang H., Li Z., Zhou B., He Y., Wang J., Aidarova S., Sarsenbekuly B. (2022). Adv. Colloid Interface Sci..

[cit31] Salager J.-L., Forgiarini A. M., Bullón J. (2013). J. Surfactants Deterg..

[cit32] Salager J.-L., Forgiarini A. M., Márquez L., Manchego L., Bullón J. (2013). J. Surfactants Deterg..

[cit33] Zhou X. L., Lee L. T., Chen S. H., Strey R. (1992). Phys. Rev. A: At., Mol., Opt. Phys..

[cit34] Frielinghaus H., Kerscher M., Holderer O., Monkenbusch M., Richter D. (2012). Phys. Rev. E: Stat., Nonlinear, Soft Matter Phys..

[cit35] Vargas-Ruiz S., Soltwedel O., Micciulla S., Sreij R., Feoktystov A., von Klitzing R., Hellweg T., Wellert S. (2016). Langmuir.

[cit36] Holderer O., Lipfert F., Frielinghaus H., Ohl M., Richter D. (2015). EPJ Web Conf..

[cit37] Kerscher M., Busch P., Mattauch S., Frielinghaus H., Richter D., Belushkin M., Gompper G. (2011). Phys. Rev. E: Stat., Nonlinear, Soft Matter Phys..

[cit38] Lipfert F., Kerscher M., Mattauch S., Frielinghaus H. (2019). J. Colloid Interface Sci..

[cit39] Berghaus M., Paulus M., Salmen P., Al-Ayoubi S., Tolan M., Winter R. (2016). J. Phys. Chem. B.

[cit40] Soprunyuk V., Reinecker M., Schranz W. (2016). Phase Transitions.

[cit41] Schemmel S. , Akcakayiran D., Rother G., Brulet A., Farago B., Hellweg T. and Findenegg G. H., MRS Online Proceedings Library, 2003, vol. 790, p. 72, 10.1557/PROC-790-P7.2

[cit42] Hellweg T., Schemmel S., Rother G., Brûlet A., Eckerlebe H., Findenegg G. H. (2003). Eur. Phys. J. E: Soft Matter Biol. Phys..

[cit43] Prause A., Hörmann A., Cristiglio V., Smales G. J., Thünemann A. F., Gradzielski M., Findenegg G. H. (2021). Mol. Phys..

[cit44] Šolcová O., Matějová L., Schneider P. (2006). Appl. Catal., A.

[cit45] Foxtrot 3.3.4, 2016

[cit46] Zhang F., Ilavsky J., Long G. G., Quintana J. P. G., Allen A. J., Jemian P. R. (2010). Metall. Mater. Trans. A.

[cit47] Wiltzius P., Bates F. S., Dierker S. B., Wignall G. D. (1987). Phys. Rev. A.

[cit48] Smarsly B., Göltner C., Antonietti M., Ruland W., Hoinkis E. (2001). J. Phys. Chem. B.

[cit49] Enke D., Janowski F., Schwieger W. (2003). Microporous Mesoporous Mater..

[cit50] Dierker S. B., Wiltzius P. (1991). Phys. Rev. Lett..

[cit51] Lin M. Y., Sinha S. K., Drake J. M., Wu X., Thiyagarajan P., Stanley H. B. (1994). Phys. Rev. Lett..

[cit52] Teubner M., Strey R. (1987). J. Chem. Phys..

[cit53] Beaucage G. (1996). J. Appl. Crystallogr..

[cit54] Mitropoulos A. C., Haynes J. M., Richardson R. M., Kanellopoulos N. K. (1995). Phys. Rev. B: Condens. Matter Mater. Phys..

[cit55] Kim M.-H., Glinka C. J. (2006). Microporous Mesoporous Mater..

[cit56] Höhr A., Neumann H. B., Schmidt P. W., Pfeifer P., Avnir D. (1988). Phys. Rev. B: Condens. Matter Mater. Phys..

[cit57] Schneider K., Verkoyen P., Krappel M., Gardiner C., Schweins R., Frey H., Sottmann T. (2020). Langmuir.

[cit58] Pizzino A., Molinier V., Catté M., Salager J.-L., Aubry J.-M. (2009). J. Phys. Chem. B.

[cit59] Glover C. J., Puerto M. C., Maerker J. M., Sandvik E. L. (1979). Soc. Pet. Eng. J..

[cit60] Tagavifar M., Herath S., Weerasooriya U. P., Sepehrnoori K., Pope G. (2018). SPE J..

[cit61] Tagavifar M., Jang S. H., Chang L., Mohanty K., Pope G. (2018). Fuel.

[cit62] Hirasaki G. J., van Domselaar H. R., Nelson R. C. (1983). Soc. Pet. Eng. J..

