# Supplementary material for: Confined microemulsions: pore diameter induced change of the phase behavior
Source: RSC Adv. 2024 Apr 19;14(18):12735–41. doi: 10.1039/d4ra01283f (PMC11027042; doi:10.1039/d4ra01283f)
Supplement: RA-014-D4RA01283F-s001 [file RA-014-D4RA01283F-s001.pdf]

Electronic Supplementary Material (ESI) for RSC Advances.
This journal is © The Royal Society of Chemistry 2024

Supporting Information

Confined Microemulsions: Pore Diameter Induced Change of the Phase Behavior

René Haverkamp,[a] Margarethe Dahl,[b] Tim Julian Stank,[a] Jessica Hübner,[c] Peter Strasser, [c] Stefan Wellert,*[b] Thomas Hellweg,*[a]

[a] René Haverkamp,Tim Julian Stank,Thomas Hellweg*, Department of Physical and Biophysical Chemistry, University of Bielefeld, Universitätsstraße 25, Bielefeld, 33615, Germany
E-mail: thomas.hellweg@uni-bielefeld.de

[b] Margarethe Dahl, Stefan Wellert, Stranski-Laboratorium für Physikalische und Theoretische Chemie, Technical University of Berlin, Straße des 17. Juni 124, Berlin, 10623, Germany
E-mail: s.wellert@tu-berlin.de

[c] Jessica Hübner, Peter Strasser, Department of Chemistry, Chemical Engineering Division, Technical University of Berlin, Straße des 17. Juni 124, Berlin, 10623, Germany
E-mail: pstrasser@tu-berlin.de

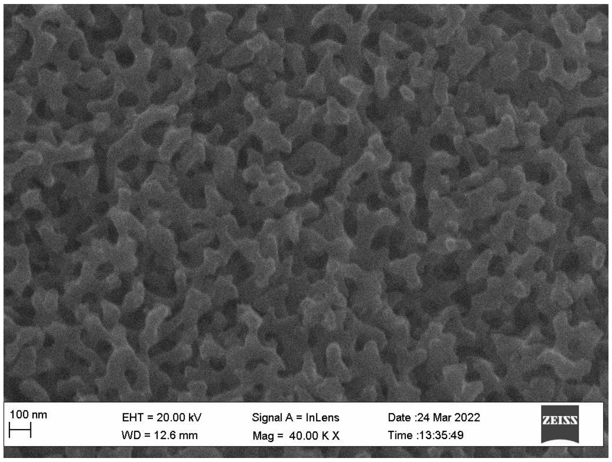

Figure 1: SEM Micrograpgh of CPG1000.

Table 1: Fit results of the Teubner-Strey model for ME (equation 5), d is the domain size, ξ, the correlation length, and B, a scaling factor.

T / °C	d / Å	ξ / Å	B
5	144	153	9885
10	163	124	3235
15	188	105	2491
20	191	97	3957
30	184	106	5197
35	181	92	3388
40	145	72	1595
45	120	61	1000
50	89	40	317
55	69	27	100

Table 2: Fit parameters of CPG184 (equation 9). The parameters of the Teubner-Strey model are d, the domain size, ξ, the correlation length, and B, a scaling factor. The parameters of the Lozentzian contribution are a scaling parameter A, the exponent n, the peak maximum q_0, and the correlation length ξ_L. The intensity of the CPG matrix is scaled by the parameter C.

T / °C	d / Å	ξ / Å	n	ξ_L / Å	B	A	C	q_0 / / Å-1
5	234	129	4	301	25199	215	1.38	0.012
10	231	133	4	301	28476	292	1.43	0.012
15	231	146	4	299	39166	342	1.44	0.012
20	240	127	4	301	39297	99	1.02	0.012
25	244	118	4	301	35520	127	0.89	0.012
30	233	117	4	299	31278	315	0.95	0.012
35	230	106	4	299	20511	424	1.08	0.012
40	230	91	4	301	13089	91	1.04	0.012
45	234	102	4	301	14337	109	1.20	0.012
50	234	112	4	301	17271	133	1.29	0.012
55	236	119	4	301	20650	122	1.29	0.012

Table 3: Fit parameters of CPG500 (equation 9). The parameters of the Teubner-Strey model are d, the domain size, ξ, the correlation length, and B, a scaling factor. The parameters of the Lozentzian contribution are a scaling parameter A, the exponent n, the peak maximum q_0, and the correlation length ξ_L. The intensity of the CPG matrix is scaled by the parameter C.

T / °C	d / Å	ξ / Å	n	ξ_L / Å	B	A	C	q_0 / / Å-1
5	154	80	4	299	1198	648	1.05	0.012
10	181	95	4	301	2461	779	1.06	0.012
15	220	93	4	301	3066	1111	1.03	0.011
20	258	92	-	-	3949	-	0.95	-
25	320	102	-	-	8302	-	0.79	-
30	236	96	-	-	4479	-	0.99	-
35	177	88	4	301	2453	464	1.05	0.011
40	147	77	4	301	1128	408	0.95	0.011
45	132	62	4	301	487	441	0.96	0.011
50	129	48	4	301	191	504	0.98	0.011
55	129	41	4	301	100	572	0.98	0.011

Table 4: Fit parameters of CPG1000 (equation 9). The parameters of the Teubner-Strey model are d, the domain size, ξ, the correlation length, and B, a scaling factor. The parameters of the Lozentzian contribution are a scaling parameter A, the exponent n, the peak maximum q_0, and the correlation length ξ_L. The intensity of the CPG matrix is scaled by the parameter C.

T / °C	d / Å	ξ / Å	n	ξ_L / Å	B	A	C	q_0 / / Å-1
5	139	125	-	-	2701	-	0.92	-
10	167	140	-	-	4877	-	1.00	-
15	204	129	-	-	5703	-	1.08	-
20	246	110	-	-	4952	-	0.97	-
25	247	133	-	-	8915	-	0.98	-
30	214	114	-	-	4979	-	1.07	-
35	168	95	-	-	2307	-	1.14	-
40	140	77	-	-	1025	-	1.19	-
45	120	64	-	-	603	-	1.19	-
50	108	54	-	-	293	-	1.21	-
55	98	46	-	-	142	-	1.18	-
